# Prophylactic inguinal lymphadenectomy for high-risk cN0 penile cancer: The optimal surgical timing

**DOI:** 10.3389/fonc.2023.1069284

**Published:** 2023-02-21

**Authors:** Shanjin Ma, Jian Zhao, Zhiwei Liu, Tao Wu, Sheng Wang, Chengwen Wu, Lei Pan, Xiaoye Jiang, Zhihao Guan, Yanjun Wang, Dian Jiao, Fengqi Yan, Keying Zhang, Qisheng Tang, Jianjun Ma

**Affiliations:** ^1^ Department of Urology, Tangdu Hospital, Fourth Military Medical University, Xi’an, China; ^2^ Department of Urology, The 955th Hospital of Army, Changdu, China; ^3^ Department of Orthopedics, Tangdu Hospital, Fourth Military Medical University, Xi’an, China; ^4^ Department of Urology, Xijing Hospital, Fourth Military Medical University, Xi’an, China

**Keywords:** penile cancer, inguinal lymphadenectomy, prophylactic dissection, timing of lymphadenectomy, delayed

## Abstract

**Background:**

Few reports have investigated the oncologically safe timing of prophylactic inguinal lymphadenectomy for penile cancer patients with clinically normal inguinal lymph nodes (cN0), particularly those who received delayed surgical treatment.

**Methods:**

The study included pT1aG2, pT1b-3G1-3 cN0M0 patients with penile cancer who received prophylactic bilateral inguinal lymph nodes dissection (ILND) at the Department of Urology of Tangdu Hospital between October 2002 and August 2019. Patients who received simultaneous resection of primary tumor and inguinal lymph nodes were assigned to the immediate group, while the rest were assigned to the delayed group. The optimal timing of lymphadenectomy was determined based on the time-dependent ROC curves. The disease-specific survival (DSS) was estimated based on the Kaplan–Meier curve. Cox regression analysis was used to evaluate the associations between DSS and the timing of lymphadenectomy and tumor characteristics. The analyses were repeated after stabilized inverse probability of treatment weighting adjustment.

**Results:**

A total of 87 patients were enrolled in the study, 35 of them in the immediate group and 52 in the delayed group. The median (range) interval time between primary tumor resection and ILND of the delayed group was 85 (29-225) days. Multivariable Cox analysis demonstrated that immediate lymphadenectomy was associated with a significant survival benefit (HR, 0.11; 95% CI, 0.02–0.57; *p* = 0.009). An index of 3.5 months was determined as the optimal cut-point for dichotomization in the delayed group. In high-risk patients who received delayed surgical treatment, prophylactic inguinal lymphadenectomy within 3.5 months was associated with a significantly better DSS compared to dissection after 3.5months (77.8% and 0%, respectively; log-rank *p*<0.001).

**Conclusions:**

Immediate and prophylactic inguinal lymphadenectomy in high-risk cN0 patients (pT1bG3 and all higher stage tumours) with penile cancer improves survival. For those patients at high risk who received delayed surgical treatment for any reason, within 3.5 months after resection of the primary tumor seems to be an oncologically safe window for prophylactic inguinal lymphadenectomy.

## 1 Introduction

Although penile cancer is a rare disease that accounts for only 1% of all malignancies in men worldwide, the mortality has risen over the past decade (1). Without timely and reasonable treatment, patients will die of complications related to local progression or metastasis within two years after diagnosis (2). Penile cancer metastasizes mainly through the lymphatic system (3). Studies have shown that the first site of penile cancer metastasis is the inguinal lymph nodes, directly affecting the survival of patients (4). According to statistics, the 5-year survival rate of penile cancer patients without inguinal lymph node transfer can be as high as 85%~100%, and the survival rate after metastasis drops to 15%~45% (5). In current treatment guidelines, inguinal lymph node dissection (ILND) has become the standard treatment for lymph node management in penile cancer (6). Penile cancer patients with palpable inguinal lymph nodes have an 80% probability of lymphatic metastasis (7, 8), and radical ILND is recommended for those patients (6, 9).

With the development of medical technology, the early diagnosis rate of penile cancer has been continuously increasing. As reported previously, 20% of clinically negative lymph nodes have micro-metastases (10), and the five-year survival rate after prophylactic ILND is as high as 80%, much higher than that of patients who receive therapeutic surgery with lymphatic metastases (2). As a result, given the significant difference in survival rate, the treatment of patients with clinically normal inguinal lymph nodes (cN0) has gradually become an important part of penile cancer diagnosis and management. However, the presence of negative postoperative lymph nodes and complications imply that some patients have undergone some degree of overtreatment, leaving prophylactic inguinal lymphadenectomy of cN0 patients controversial (11). In addition, many patients receive delayed inguinal lymphadenectomy due to the referral process or personal indecision about surgical treatment. Therefore, it is vitally important to assess the timing of prophylactic surgical treatment.

According to EAU guidelines, invasive lymph node staging, which can be done by either dynamic sentinel-node biopsy (DSNB) or by modified ILND, is recommended for intermediate- and high risk cN0 penile cancer (6). However, given the low prevalence of DSNB technology, together with the high morbidity of complications of radical ILND, modified ILND is still the optimal choice for clinical staging and treatment of intermediate- and high-risk cN0 penile cancer.

To our knowledge, due to the lack of high-grade clinical studies, there is no unified opinion on the timing of prophylactic ILND for cN0 penile cancer patients, and the influence of the time interval between primary tumor resection and ILND on prognosis is not yet clear.

Our objectives were to investigate the association between the timing of prophylactic inguinal lymphadenectomy and postoperative survival and to identify a relatively safe time window for cN0 patients with penile cancer, especially those who received delayed ILND.

## 2 Patients and methods

### 2.1 Study populations

We conducted a retrospective, single-center cohort study of cN0 penile cancer patients receiving specialist treatment at the Department of Urology of Tangdu Hospital (Xi’an, China) between October 2002 and August 2019. The inclusion and exclusion criteria were as follows. All patients who were diagnosed with pT1aG2, pT1b-3G1-3 cN0M0 squamous cell carcinoma of the penis and underwent bilateral modified ILND were included in the present analysis to obtain a homogenous study population. Node-negative patients were defined as those who had no clinically palpable inguinal lymph nodes at presentation, nor did the lymph nodes visibly enlarge during imaging examination. Patients were excluded if they were simultaneously diagnosed with other malignant tumors, received neoadjuvant chemotherapy, presented with palpable adenopathy at the time of surgery, or were missing data on follow-up and survival. Current guidelines recommend neoadjuvant chemotherapy followed by surgery or palliative radiotherapy for patients with T4 diseases, especially those with locally advanced and ulcerated cases (6); unresectable primary tumors, bulky inguinal adenopathy, and pelvic lymphadenopathy may benefit from neoadjuvant systemic chemotherapy (12, 13). To eliminate bias during histopathological examination and prognosis of prophylactic dissection, patients with T4 diseases who received neoadjuvant chemotherapy or radiotherapy were excluded.

The study was approved by the Institutional Review Board of Tangdu Hospital, Fourth Military Medical University (No: TDLL-KY-202104-01), and informed consent was exempted.

### 2.2 Treatment protocol

Patients who received simultaneous resection of primary tumor and inguinal lymph nodes were assigned to the immediate group, while other patients were assigned to the delayed group. No palpable or visibly enlarged inguinal lymph nodes when they received prophylactic surgical treatment. The cases were treated in accordance with modern treatment protocol, including standard preoperative imaging, primary tumor treatment, and standard surgical templates. Nodal staging was accomplished by physical examination and imaging. Inguinal computed tomography (CT) examination was used in obese patients in whom palpation was unreliable to exclude lymph nodes enlargement. To other patients, a physical examination of both groins was performed in order to record the number, laterality, and characteristics of inguinal nodes. If nodes were not palpable, inguinal B-ultrasound was performed first.

All these following boundaries constituted the extent of modified ILND: the spermatic cord formed the upper boundary, and the fossa ovalis formed the lower boundary; the inner and outer boundaries were the lateralis of the long adductor muscle and the femoral artery, respectively. Compared with radical ILND, the modified procedure decreased the length of the skin incision and the scope of dissection, preserved the saphenous vein and fascia lata, and avoided the transposition of the sartorius muscle, decreasing morbidity related to groin dissection (14–16).

Before 2015 all procedures were open ILNDs, and since 2015 we have performed video-endoscopic ILND. Additional ipsilateral pelvic LND (pLND) (external iliac and obturator) was performed if two or more inguinal lymph nodes were involved after ILND and adjuvant chemotherapy was provided. The Clavien-Dindo classification system was used to judge operation-related complications.

### 2.3 Outcomes and definitions

The primary endpoint was disease-specific survival (DSS), which was calculated from the date of ILND to date of penile cancer-related death during follow-up. Pretreatment variables used for analysis included age, stage and pathologic grade of the primary tumor, lymph node stage (clinical and pathological), lymphovascular invasion (LVI), and timing of lymphadenectomy.

### 2.4 Follow-up

All patients were followed up for at least two years (the patients who died within two years after operation were followed up to the time of death). The discharged patients were followed up every three months before two years; the patients with pN0 stage tumors were followed up every 12 months from three to five years; and the patients with pN+ stage tumors were followed up every six months from three to five years to evaluate recurrence, distant metastasis, and survival in the groin region. The main methods of follow-up examination include physical examination, B-ultrasound, and CT/MRI. The follow-up data were collected by consulting outpatient revisit records, telephone follow-up, and so on.

### 2.5 Statistical analyses

Descriptive analysis was performed after testing for normality and homogeneity of variance for continuous variables in the baseline data of the entire cohort of patients. Normally distributed variables were reported as mean ± standard deviation, while non-normally distributed variables were expressed as median (interquartile range), and comparisons between groups were conducted using independent sample t test or nonparametric test (Mann–Whitney U test). Categorical variables were expressed as the frequency (%), and differences between groups were compared using chi-square test, Fisher’s exact test, or nonparametric test (Mann–Whitney U test).

The standardized mean difference (SMD) was calculated to assess the uniformity of the distribution of baseline characteristics. A propensity score was obtained by fitting a logistic regression model based on confounding factors such as age, the primary tumor stage, grade, and LVI. The inverse probability of treatment weighting (IPTW) method based on the propensity score was used to create a virtual sample that satisfied the balance of baseline characteristics and to control the differences in confounding factors between the two groups. The increased sample size after weighting could easily lead to false-positive results; thus, stabilized IPTW (S-IPTW) was applied to make the virtual weighted sample size close to the original. The false-positive rate and the rate of type I errors were reduced to balance the confounding factors between groups to the greatest extent possible.

The Kaplan–Meier method was used to describe the five-year DSS, and log-rank test was used to compare the differences between the survival curves. Cox regression was used to analyze the independent factors of the primary end point, and variables with *p* < 0.05 in univariate analysis or with clinical importance were included in a multivariate analysis. Time-dependent receiver operating characteristic (ROC) curves were drawn based on the surgical delay time and the survival outcomes in the delayed group to determine the optimal cut-off point according to the Youden index.

The *p* values were derived from two-tailed tests, and all differences were considered statistically significant at *p*<0.05. The Statistical Package for the Social Sciences software (version 26.0) and R program (version 3.6.0) were used for all statistical analysis.

## 3 Results

### 3.1 Study populations

**Figure 1:**
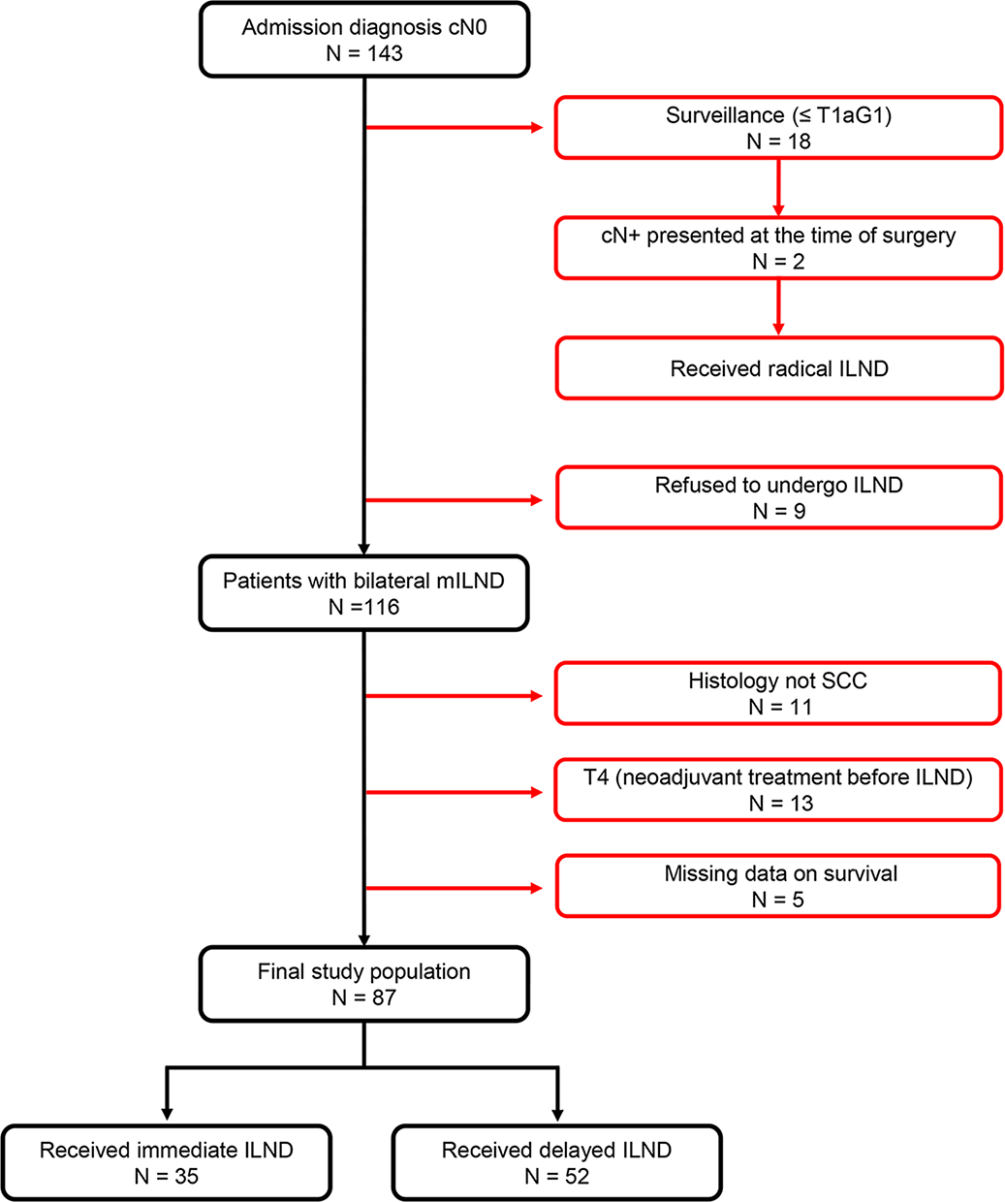
Patient enrollment flowchart.

Of 143 consecutive penile cancer patients with cN0 diseases who were admitted to Tangdu Hospital and received related treatment between October 2002 and August 2019, a total of 56 patients were excluded from the present study because they had non- squamous cell carcinoma histology, underwent surveillance (≤pT1aG1), refused to receive ILND, were missing survival data, or had T4 diseases. Thus, 87 (61%) patients were included in the final analysis, of whom 35 and 52 received immediate and delayed ILND as a primary treatment modality, respectively (**Figure 1**).

### 3.2 Baseline characteristics

Over a median follow-up of 43 (38, 52.5) months, 19.5% of patients had a primary end-point event (17 patients died, of whom two and 15 underwent immediate and delayed ILND, respectively). **Table 1** shows the demographic and clinical characteristics of 87 patients with no inguinal disease presented at the time of ILND. The median (interquartile range [IQR]) age of the cohort was 52 (49, 59) years. Overall, 43.7% and 56.3% of patients were at intermediate and high risk of lymphatic spread, respectively. Tumor-positive inguinal nodes were found in 34 of the 87 patients (39.1%) after ILND, and only 21 of those with pN+ disease received subsequent pLND with adjuvant chemotherapy. A total of 18.4% of the patients experienced an inguinal recurrence during the follow-up period.

**Table 1:** Baseline patient and tumor characteristics in 87 cN0 penile cancer patients.

Median mos follow-up (IQR)	43	(38, 52.5)
Median yrs age (IQR)	52	(49, 59)
No. tumor stage (%)		
pT1a	20	(23.0)
pT1b	28	(32.2)
pT2	27	(31.0)
pT3	12	(13.8)
No. tumor grade (%)		
G1	21	(24.1)
G2	43	(49.4)
G3	23	(26.4)
No. lymphovascular invasion (%)	43	(49.4)
No. risk level (%)		
Intermediate-risk	38	(43.7)
High-risk	49	(56.3)
No. timing of ILND (%)		
Immediate	35	(40.2)
Delayed	52	(59.8)
No. pathologic nodes status (%)		
pN0	53	(60.9)
pN1	15	(17.2)
pN2	16	(18.4)
pN3	3	(3.4)
No. pLND (%)	21	(24.1)
No. adjuvant chemotherapy (%)	21	(24.1)
No. inguinal recurrence (%)	16	(18.4)

IQR, inter-quartile range; ILND, inguinal lymph node dissection; pLND, pelvic lymph node dissection.

The distribution of the patients’ baseline characteristics according to the timing of ILND is shown in **Table 2** for both the unweighted and weighted samples. In the IPTW sample, 84 patients received immediate ILND, and 88 received delayed ILND. In the S-IPTW sample, 34 and 52 patients underwent immediate and delayed ILND, respectively. The highest SMD value in the weighted data was 0.133 for the tumor stage; all other baseline variables had SMD values less than 0.1. Two weighted samples achieved adequate balance between the immediate and delayed groups for all covariates, with differences attenuated (**Figure S1**).

**Table 2:** Descriptive characteristics of 87 patients who underwent either immediate ILND or delayed ILND, with unweighted and S-IPTW adjustments.

Variables	Unweighted			S-IPTW		
	Immediate	Delayed	*p*	Immediate	Delayed	*p*
n	35	52		33.78	52.34	
Age, yrs (median [IQR])	54.86 (8.11)	52.79 (6.93)	0.206	53.23 (7.54)	53.36 (6.67)	0.932
Tumor stage, n (%)			0.955			0.951
pT1a	9 (25.7)	11 (21.2)		8.0 (23.7)	11.9 (22.7)	
pT1b	11 (31.4)	17 (32.7)		13.3 (39.4)	17.9 (34.2)	
pT2	10 (28.6)	17 (32.7)		8.4 (24.7)	15.3 (29.2)	
pT3	5 (14.3)	7 (13.5)		4.1 (12.1)	7.3 (13.9)	
Tumor grade, n (%)			0.068			0.990
G1	11 (31.4)	10 (19.2)		8.4 (25.0)	13.1 (25.0)	
G2	12 (34.3)	31 (59.6)		16.1 (47.6)	25.6 (49.0)	
G3	12 (34.3)	11 (21.2)		9.3 (27.4)	13.6 (26.1)	
LVI, n (%)	14 (40.0)	29 (55.8)	0.221	16.8 (49.7)	26.3 (50.2)	0.970

### 3.3 Survival analyses

**Figure 2:**
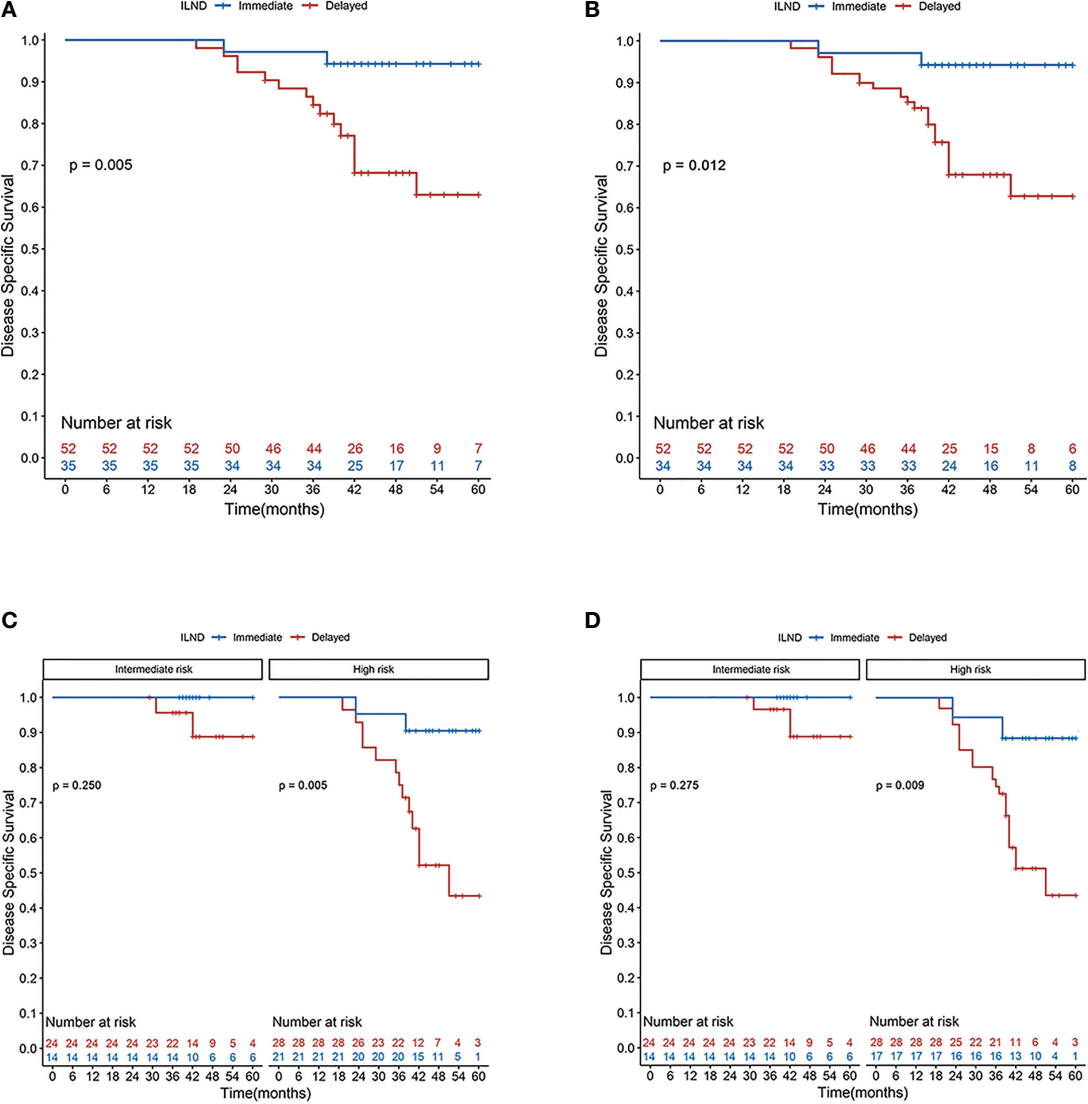
Disease-specific survival rates according to Kaplan–Meier curves in patients undergoing immediate (blue line) and delayed (red line) inguinal lymph node dissection: **(A)** primary cohort; **(B)** cohort after S-IPTW adjustment; **(C)** primary cohort stratified by risk level; and **(D)** cohort stratified by risk level after S-IPTW adjustment.

In the unweighted sample, Kaplan–Meier analysis and log-rank testing, stratified by the timing of ILND, revealed a statistically significant difference in DSS between the two groups. Patients in the immediate dissection group demonstrated a higher five-year DSS (94.3% vs. 63.0%) for delayed dissection with an unadjusted hazard ratio (HR) of 0.16 (95% confidence interval [CI], 0.04–0.70). Multivariable Cox analysis demonstrated immediate ILND was associated with a significant DSS benefit (HR, 0.11; 95% CI, 0.02–0.57; *p* = 0.009). Subgroup analysis by risk level showed that in the high-risk group, the five-year DSS was 90.5% for immediate ILND compared to 43.5% for delayed dissection (log-rank *p* = 0.005). In contrast, five-year DSS in intermediate-risk patients was not significantly improved by immediate ILND compared to delayed dissection (100% vs. 88.8%, respectively; log-rank *p* = 0.250). We observed no significant associations between age, tumor stage, G1 and G2, or LVI with five-year DSS. In S-IPTW analysis, however, the results were somewhat different. Based on the results of weighted univariate analysis, the multivariable Cox regression was adjusted for age but not for tumor stage. In addition, LVI was significantly associated with decreased five-year DSS (HR, 3.59; 95% CI, 1.04–12.42; *p* = 0.044; **Table 3**; **Figure 2**).

**Table 3:** Univariate and multivariable Cox regression in 87 cN0 penile cancer patients who underwent either immediate ILND or delayed ILND, with unweighted and S-IPTW adjustments.

Variables	Univariate Analysis		Multivariable Analysis	
	HR (95%CI)	*p*	HR (95%CI)	*p*
Unweighted Cox Regression				
Age	1.040(0.977-1.107)	0.219		
Tumor stage				
T1a	Reference		Reference	
T1b	1.353(0.123-14.920)	0.805	0.205(0.011-3.813)	0.288
T2	7.634(0.967-60.270)	0.054	1.421(0.089-22.781)	0.804
T3	8.788(1.026-75.270)	0.047*	2.061(0.128-33.087)	0.610
Tumor grade				
G1	Reference		Reference	
G2	1.602(0.323-7.941)	0.564	1.169(0.181-7.526)	0.870
G3	5.237(1.131-24.259)	0.034*	6.732(1.175-38.569)	0.032*
Lymphovascular invasion	5.286(1.519-18.400)	0.009*	4.053(0.749-21.939)	0.104
Immediate ILND	0.159(0.036-0.697)	0.015*	0.107(0.020-0.571)	0.009*
S-IPTW Cox Regression				
Age	1.065(1.002-1.132)	0.042	1.073(0.997-1.156)	0.061
Tumor stage				
T1a	Reference			
T1b	1.084(0.099-11.900)	0.947		
T2	6.552(0.880-48.760)	0.066		
T3	7.247(0.966-54.350)	0.054		
Tumor grade				
G1	Reference		Reference	
G2	1.032(0.208-5.112)	0.969	0.678(0.124-3.724)	0.655
G3	4.958(1.107-22.203)	0.036*	3.795(0.813-17.705)	0.090
Lymphovascular invasion	3.649(1.057-12.590)	0.041*	3.589(1.037-12.421)	0.044*
Immediate ILND	0.160(0.035-0.737)	0.019*	0.129(0.023-0.706)	0.018*

S-IPTW, stabilized inverse probability of treatment weighting; ILND, inguinal lymph node dissection; HR, hazard ratio; CI, confidence internal. *p* values are derived from two-tailed tests. *All differences statistically significant at *p<0.05*.

### 3.4 Selection of optimal time to ILND

Based on the maximum sensitivity and specificity, the optimal cut-off points for the three-, four-, and five-year ROC curves were all 105 days after primary tumor resection (areas under the curve of 0.87, 0.90, and 0.81, respectively; **Figure S2**). ILND within 3.5 months was classified as early dissection (n = 34), while ILND after 3.5 months was classified as late dissection (n = 18).

### 3.5 Subgroup analysis of patients with delayed ILND

**Figure 3:**
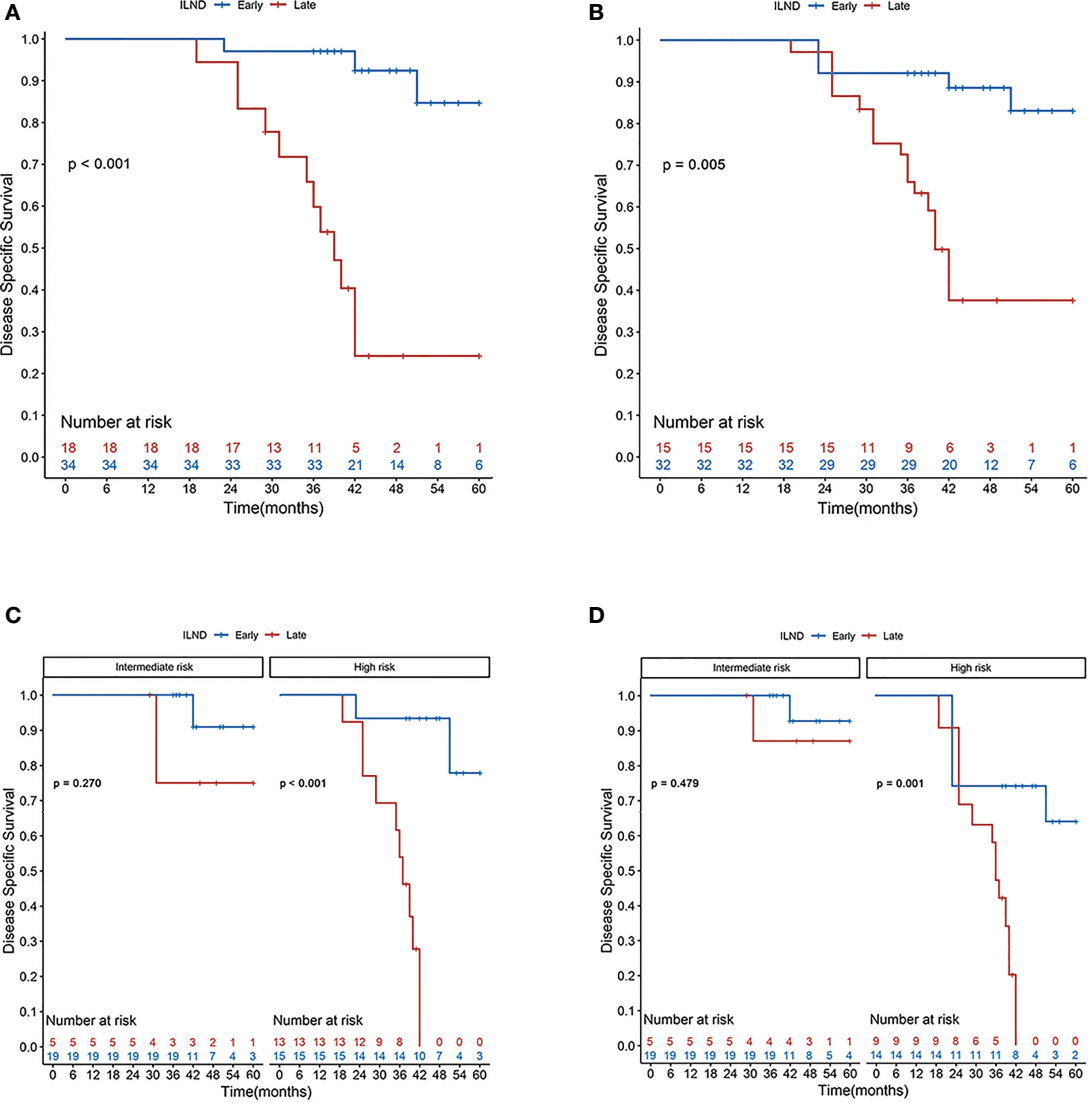
Disease-specific survival rates according to the Kaplan–Meier curves in patients undergoing early (blue line) and late (red line) inguinal lymph node dissection: **(A)** primary cohort; **(B)** cohort after S-IPTW adjustment; **(C)** primary cohort stratified by risk level; and **(D)** cohort stratified by risk level after S-IPTW adjustment.

Distributions of tumor grade and LVI of the two new classified groups were with statistical difference. After IPTW and S-IPTW adjustments, the two groups achieved adequate balance for all covariates (all *p*>0.05; **Table 4**). When comparing the early and late ILND groups, which had five-year DSS values of 84.7% and 24.2%, respectively, this 3.5 month cut-point was statistically significant (log-rank *p*<0.001). Based on subgroup analysis by risk level, in high-risk patients, early ILND was associated with a significantly higher five-year DSS compared to deferred dissection (77.8% and 0%, respectively; log-rank *p*<0.001). In contrast, early ILND did not statistically improve the five-year DSS compared to delayed dissection among intermediate-risk patients (90.9% and 75.0%, respectively; log-rank *p* = 0.270). Univariate and multivariable Cox regressions demonstrated that early ILND was associated with a significant increase in DSS (HR, 0.07; 95% CI, 0.02–0.27; *p*<0.001). Similar results were obtained in the S-IPTW samples (**Table 5**; **Figure 3**).

**Table 4:** Descriptive characteristics of 52 patients who underwent either early ILND or late ILND, with unweighted and S-IPTW adjustments.

Variables	Unweighted Sample			S-IPTW		
	Early	Late	*p*	Early	Late	*p*
n	34	18		31.88	14.93	
Age, yrs (median [IQR])	52.21 (6.83)	53.89 (7.19)	0.410	53.18 (7.03)	53.82 (7.63)	0.799
Tumor stage, n (%)			0.127			0.703
pT1a	9 (26.5)	2 (11.1)		7.2 (22.6)	3.8 (25.7)	
pT1b	12 (35.3)	5 (27.8)		10.2 (32.1)	4.2 (28.0)	
pT2	11 (32.4)	6 (33.3)		12.4 (39.0)	4.4 (29.4)	
pT3	2 (5.9)	5 (27.8)		2.0 (6.3)	2.5 (16.9)	
Tumor grade, n (%)			0.033*			0.588
G1	9 (26.5)	1 (5.6)		6.6 (20.7)	1.1 (7.6)	
G2	21 (61.8)	10 (55.6)		18.2 (57.1)	10.0 (66.7)	
G3	4 (11.8)	7 (38.9)		7.1 (22.2)	3.8 (25.7)	
LVI, n (%)	15 (44.1)	14 (77.8)	0.042*	16.9 (52.9)	8.8 (59.0)	0.732

**Table 5:** Univariate and multivariable Cox regression in 52 cN0 penile cancer patients who underwent either early ILND or late ILND, with unweighted and S-IPTW adjustments.

Variables	Univariate Analysis		Multivariable Analysis	
	HR (95%CI)	*p*	HR (95%CI)	*p*
Unweighted Cox Regression				
Age	1.082 (1.004-1.167)	0.039*	1.072 (0.988-1.163)	0.097
Tumor stage				
T1a	Reference			
T1b	1.241 (0.133-13.70)	0.860		
T2	5.951 (0.744-47.62)	0.093		
T3	7.366 (0.819-66.27)	0.075		
Tumor grade				
G1	Reference		Reference	
G2	1.091 (0.220-5.411)	0.915	0.230 (0.030-1.743)	0.155
G3	6.616 (1.326-33.01)	0.021*	1.616 (0.217-12.03)	0.639
Lymphovascular invasion	3.510 (0.990-12.44)	0.052	2.266 (0.473-10.85)	0.306
Early ILND	0.075 (0.020-0.274)	IV0.001*	0.073 (0.016-0.323)	<.001*
S-IPTW Cox Regression				
Age	1.117 (1.014-1.250)	0.026*	1.093 (0.995-1.200)	0.063
Tumor stage				
T1a	Reference			
T1b	1.498 (0.132-17.07)	0.745		
T2	7.311 (0.864-61.89)	0.068		
T3	7.411 (0.876-62.71)	0.066		
Tumor grade				
G1	Reference			
G2	0.725 (0.160-3.292)	0.677		
G3	3.763 (0.670-21.13)	0.132		
Lymphovascular invasion	2.504 (0.653-9.601)	0.181	2.459 (0.609-9.920)	0.206
Early ILND	0.166(0.032- 0.852)	0.031*	0.195 (0.039-0.984)	0.048*

S-IPTW, stabilized inverse probability of treatment weighting; ILND, inguinal lymph node dissection; HR, hazard ratio; CI, confidence internal. *p* values are derived from two-tailed tests. *All differences statistically significant at *p<0.05*.

### 3.6 Surgical outcomes

As showed in **Tables S1**, **S2**, there was no statistically significant difference in the number of removed lymph nodes, either between patients in the immediate and the delayed groups, or in the early and the late groups. However, there were more positive lymph nodes in the delayed group than that in the immediate group, and more in the late group than that in the early group (*p*<0.001).

Overall, the complication of the whole cohort as 13.8% (4%Clavien I; 4.6%Clavien II; 5.2% Clavien III). Of the 74 (37 patients) video-endoscopic ILNDs performed in our study, only 3 surgical wounds developed operation-related complications (2 wound infections, 1 lymphorrhea), without any statistical significance. Due to the low morbidity of postoperative complications of minimally invasive surgery, we found that the results would be more accurate if we only analyze the complications of open ILND. Thus, **Table S3**, based on open ILND, showed that the immediate group had lower morbidity of wound-related complications such as wound infection and lymphorrhea than the delayed, particularly in Clavien IIIa(*p*=0.033). In addition, patients in the early group had lower morbidity of nearly all wound complications in the study and Clavien IIIa and IIIb, compared to the late group in which patients received ILND 3.5 months after primary tumor resection (**Table S4**).

## 4 Discussion

This study demonstrates that in cN0 penile cancer patients at high risk of lymphatic spread, immediate ILND after primary tumor resection is beneficial in terms of the five-year DSS. In addition, among high-risk patients (pT1G3 and all higher stage tumours) who received delayed resection for personal or medical reasons, 3.5 months is an oncologically safe window for the surgical management of inguinal lymph nodes.

Based on histological analysis, Protzel et al. found that 50% of clinically positive lymph nodes had no pathological diagnosis of metastasis after surgery (10), indicating that a considerable proportion of patients with cN0 diseases received excessive surgical treatment. In our study, 53 (60.9%) patients were pathology stage pN0 and subsequently received regular surveillance, a slightly lower percentage than that reported by Nazzani et al. (17) (68.8%; 11 of 16 patients with cN0 diseases who underwent prophylactic ILND had a postoperative pathologic diagnosis of pN0). However, although controversies still exist (11, 18, 19), especially in the timing of dissection, increasing evidence suggests that prophylactic lymph node interventions are beneficial for survival. Among clinically negative lymph nodes, 20% were reported to present micro-metastasis (10). The rate of lymphatic micro-metastasis in our study was close to 40%, which may be due to the exclusion of some pN0 patients in the study design. Notably, early ILND in node-negative patients provided superior overall survival compared with therapeutic ILND in those with regional nodal recurrence. Those cN0 patients who received prophylactic dissection had a higher five-year survival rate compared with cN+ patients (80%–90% vs. 30%–40%) (18, 20). Therefore, prophylactic ILND plays an important role in improving the postoperative survival of patients.

Our results indicated that immediate ILND was associated with better postoperative survival compared with delayed dissection. The five-year DSS of patients with immediate dissection was 94.3%, significantly higher than that (63.0%) in the delayed dissection group (log-rank *p* = 0.005). To reduce potential bias between the two groups, the data were weighted by IPTW, and S-IPTW was then performed. The adjusted results also demonstrated the survival benefit of immediate dissection, consistent with the conclusions of relevant studies (2, 20). Thus, our study provides a prognostic and survival basis for the advantages of prophylactic immediate ILND in patients with cN0 penile cancer.

In our study, risk stratification for lymphatic micro-metastasis was performed based on stage, grade, and the presence of LVI in the primary tumor. The risk categories were as follows: 1) intermediate risk, pT1aG2 and pT1bG1-2; and 2) high risk: pT1G3 and all higher stage tumours. Notably, pT1a tumors have a low risk of metastasis (11%) (21, 22), and patients with pT1a and G1 diseases can opt for surveillance. However, the optimal management strategy for the lymph nodes of patients with G2 diseases are unclear at present. For these patients, the European Association of Urology (EAU) guidelines recommend invasive lymph node staging, while the NCCN guidelines recommend surveillance. In this study, a total of 38 enrolled patients were at intermediate risk of lymph node metastasis, and all received prophylactic bilateral ILND. Among the 20 patients with pT1aG2 diseases, three patients (15%) had inguinal lymph node metastasis diagnosed by postoperative pathology, slightly higher than the overall metastasis rate in patients with pT1a tumors. Considering that tumor grade was recognized as an independent risk factor for inguinal lymphatic metastasis, we recommend prophylactic ILND for cN0 patients with pT1aG2 diseases.

As mentioned above, immediate ILND provided a certain survival advantage to patients with cN0 diseases. However, this advantage was not obvious in the intermediate-risk group; although the weighted five-year DSS of intermediate-risk patients who received immediate dissection (100%) was slightly higher than that in the delayed group (88.4%), the difference was not statistically significant (*p* = 0.275). Considering the small sample size, the conclusions about patients at intermediate risk have yet to be verified; this provides direction for a following multi-center, large-scale prospective study.

Early ILND has been shown to have a positive influence on tumor control, DSS, and recurrence-free survival in penile cancer (2, 23–25). Early dissection has been defined as ILND performed within six weeks after primary tumor resection (26). In 2017, Chipollini et al. (27) first reported an association between regional recurrence and time to lymphadenectomy. They found that three months seems to be an oncologically safe window for performing ILND because further delay reduces the recurrence-free survival rate. In our study, the classification of delayed surgical timing was based on the postoperative DSS, and the time-dependent ROC curve indicated that 3.5 months was the optimal cut-off point. The five-year DSS of cN0 patients who underwent ILND within 3.5 months was 84.7%, much higher than that of patients who received surgery after 3.5 months (24.2%; log-rank *p* < 0.001). However, in the subgroup analysis based on risk stratification in the delayed group, the timing of surgery had no statistically significant effect on survival in the intermediate-risk group (90.9% and 75.0%, respectively; log-rank *p* > 0.05), and the results were the same in the adjusted samples. Therefore, we concluded that preoperative risk stratification is of great importance in the formulation of treatment plans. In the report of Marilin et al., 17% of surveyed surgeons still opted for anti-infective treatment for clinically suspicious lymph nodes in the groin region (≤ 2 cm) before surgery (28). The EAU guidelines, however, point out that for patients with palpable unilateral or bilateral inguinal lymph nodes (cN1/cN2), the possibility of lymphatic metastasis remains high, and considering these palpable nodes as inflammatory enlargement and giving priority to antibiotics for anti-infective treatment is unreliable. Resulting in surgical treatment delayed, anti-infective treatment is not recommended as a measure to exclude the presence of micro-metastases (6).

For patients with recurrence after previous surgery, the guidelines recommend neoadjuvant chemotherapy before salvage surgery (6, 9). In this study, three patients in the immediate group received neoadjuvant chemotherapy before the second operation, and two of them survived. Meanwhile, 13 patients in the delayed group received neoadjuvant chemotherapy, and one of them survived. There was no significant difference in survival between the two groups (*p* = 0.071), and the effect of neoadjuvant chemotherapy on the prognosis was negligible in this study. In addition, patients with pN2 and pN3 diseases have poor prognosis after ILND, and EAU guidelines recommend adjuvant chemotherapy for these patients (6). In this study, three patients in the immediate group received pLND and adjuvant chemotherapy, and one of them survived, whereas 6 of 18 patients in the delayed group survived. There was no statistical difference in survival between the two groups (*p* = 1.000). Thus, in this study, the bias introduced by receiving pLND and adjuvant chemotherapy after ILND was considered to be negligible. Regarding the effect of adjuvant chemotherapy on survival after ILND, the treatment options offered by most studies are based on small-size retrospective studies or single-center studies; thus, the level of evidence is low, and no consensus has been reached on the recommended treatment. The multicenter study of Necchi et al. (13) did not find a significant survival improvement in patients receiving adjuvant chemotherapy after ILND, although subgroup analysis may show a benefit for pN3 patients with pelvic lymph node involvement. However, related studies showed that adjuvant chemotherapy improved postoperative survival (29, 30). In addition, the panel of the EAU Penile Cancer Guidelines noted that adjuvant radiotherapy in the groin region of patients with pathologically positive lymph nodes did not provide a survival benefit and therefore was not recommended as part of standard clinical care (31). In general, as mentioned above, most of the patients who need adjuvant chemotherapy are those with inguinal lymph node metastasis and pN2 and pN3 diseases, while lymphatic micro-metastasis depends more on the patient’s preoperative tumor stage, grade, and LVI. Therefore, to provide the best adjuvant therapy for patients with advanced penile cancer (≥ pN2), more prospective and international multi-center studies with large sample sizes are needed.

DSNB technique can provide accurate nodal staging while reducing the morbidity of postoperative complications (32).Overall, the morbidity of complications of DSNB is approximately 7% (33), lower than that of our study(13.8%). In addition, the sensitivity of DSNB for detecting inguinal lymph node micro-metastases in cN0 penile cancer is 88~90% (34), and the false negative rates are 4~12% (33, 35, 36). In our study, 4 (7.5%) patients with postoperative diagnosis of pN0 relapsed during follow-up, making the false negative rate similar to that of DSNB. In fact, however, the technique of DSNB is relatively complex and has a long learning curve, which involves multidisciplinary involvement in surgery, nuclear medicine, pathology and other disciplines (35, 37, 38). This puts forward high professional requirements for medical staff in terms of patient selection, pre-biopsy examination and the application of biopsy techniques. Unfortunately, there are currently few medical centers that can perform DSNB, and systematic, effective, multidisciplinary training is necessary for the widespread application of (32). Compared with radical ILND, the modified procedure decreased the length of the skin incision and the scope of dissection, preserved the saphenous vein and fascia lata, and avoided the transposition of the sartorius muscle, decreasing morbidity related to groin dissection (14–16). Modified ILND can be used as a staging procedure for penile cancer, as well as a prophylactic surgical treatment, and can significantly reduce postoperative complications. In addition, it reduces interference with the lymphatic system in the inguinal region, reducing lymphatic-related complications to 10~36% (39, 40).

Regarding the surgical methods of ILND (open vs. minimally invasive surgery), Ma et al. (41) found no statistically significant difference in tumor control and short-term survival for penile cancer. Therefore, the bias introduced by the surgical approach was considered to be negligible in this study. In this study, a total of 52 patients underwent delayed ILND, 32 of whom were referred from another hospital, and 20 newly diagnosed patients in our hospital were delayed because of hesitancy about whether to receive surgery. Although prospective studies are needed for validation, the 3.5-month operative time window provides a reference for medical centers when formulating diagnosis and treatment plans for cN0 penile cancer.

We recognize the limitations of this single-center retrospective study. First, due to the low incidence of penile cancer, the small sample size and the short follow-up time weakened the prognostic significance of the DSS-based results to a certain extent. Second, patients at intermediate and high risk who refused to receive surgery were not included in the analysis, which influenced the results to a certain degree. Third, differences in the detection and diagnosis of preoperative pathological features along with the surgical techniques of surgeons may have led to incalculable differences that affected the results. Therefore, multi-center prospective studies with large sample sizes and long-term detailed follow-up are needed.

In summary, cN0 penile cancer patients at high risk of inguinal lymphatic metastasis (pT1G3 and all higher stage tumours) benefit from simultaneous resection of the primary tumor and inguinal lymph nodes. More importantly, 3.5 months seems to be a relatively oncologically safe time window for these high-risk cN0 patients if the prophylactic inguinal lymphadenectomy is delayed for any reason.

## Data availability statement

The original contributions presented in the study are included in the article/**Supplementary Material**. Further inquiries can be directed to the corresponding authors.

## Ethics statement

The studies involving human participants were reviewed and approved by Institutional Review Board of Tangdu Hospital of Fourth Military Medical University. Written informed consent for participation was not required for this study in accordance with the national legislation and the institutional requirements.

## Author contributions

JM, QT, and KZ: Conceptualization, study design and project administration. SM, XJ, DJ, and FY: Material and data collection. SM, JZ, ZL, TW, ZG, and YW: Data analysis and interpretation. SW, CW, and LP: Formal analysis, literature review and tables. All authors have participated to draft the manuscript and revised it critically. All authors contributed to the article and approved the submitted version.

## Supplementary material

Supplementary Figure 1 SMD values of the baseline information for the primary and adjusted cohorts.

Supplementary Figure 2 Time-dependent ROC curves based on the timing of surgery in the delayed group.
