# Supplementary figures and images for: Prophylactic inguinal lymphadenectomy for high-risk cN0 penile cancer: The optimal surgical timing
Source: Front Oncol. 2023 Feb 21;13:1069284. doi: 10.3389/fonc.2023.1069284 (PMC9989449; doi:10.3389/fonc.2023.1069284)

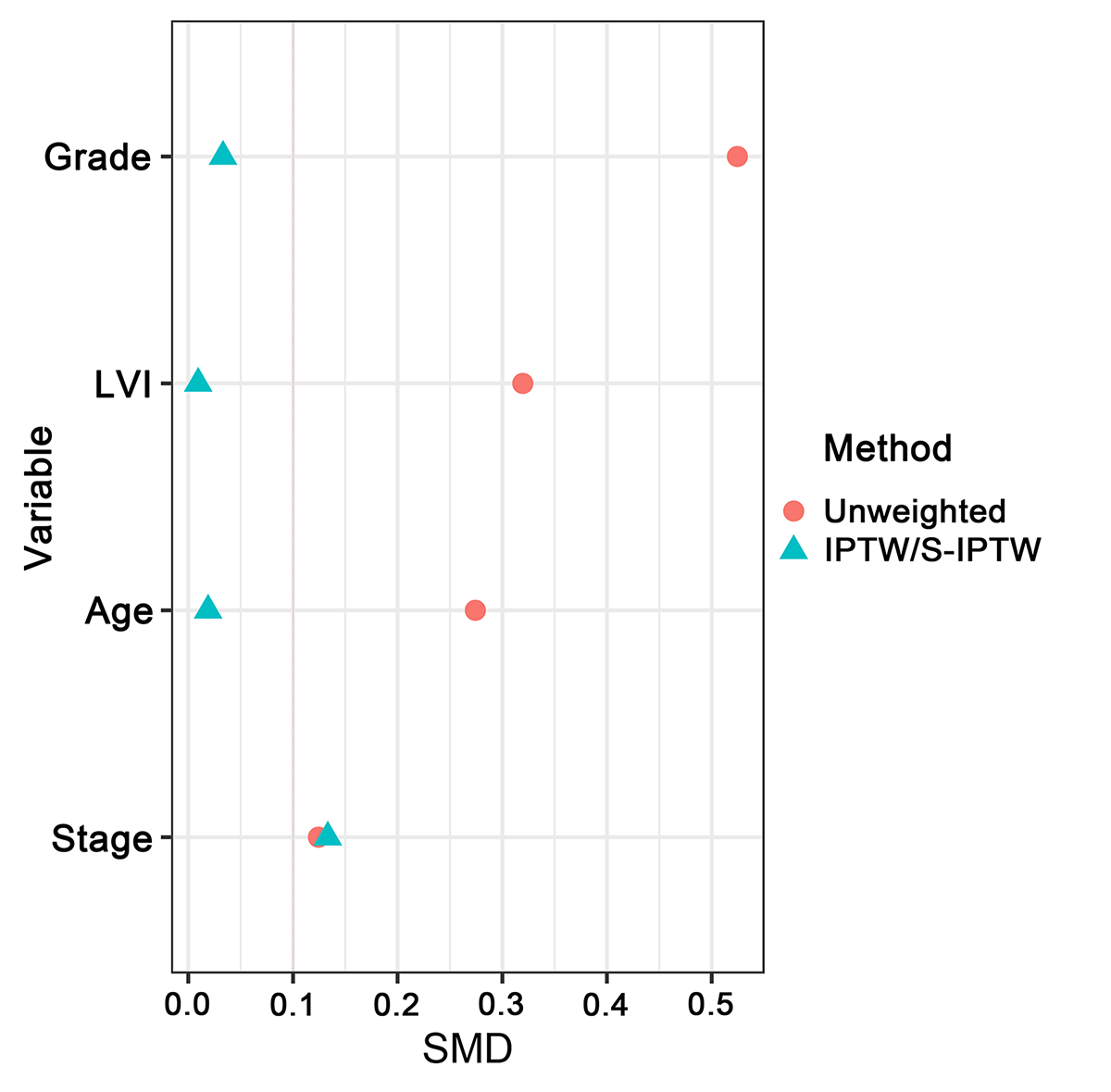

Supplement: Supplementary Figure 1 — SMD values of the baseline information for the primary and adjusted cohorts. [file Image_1.tif]

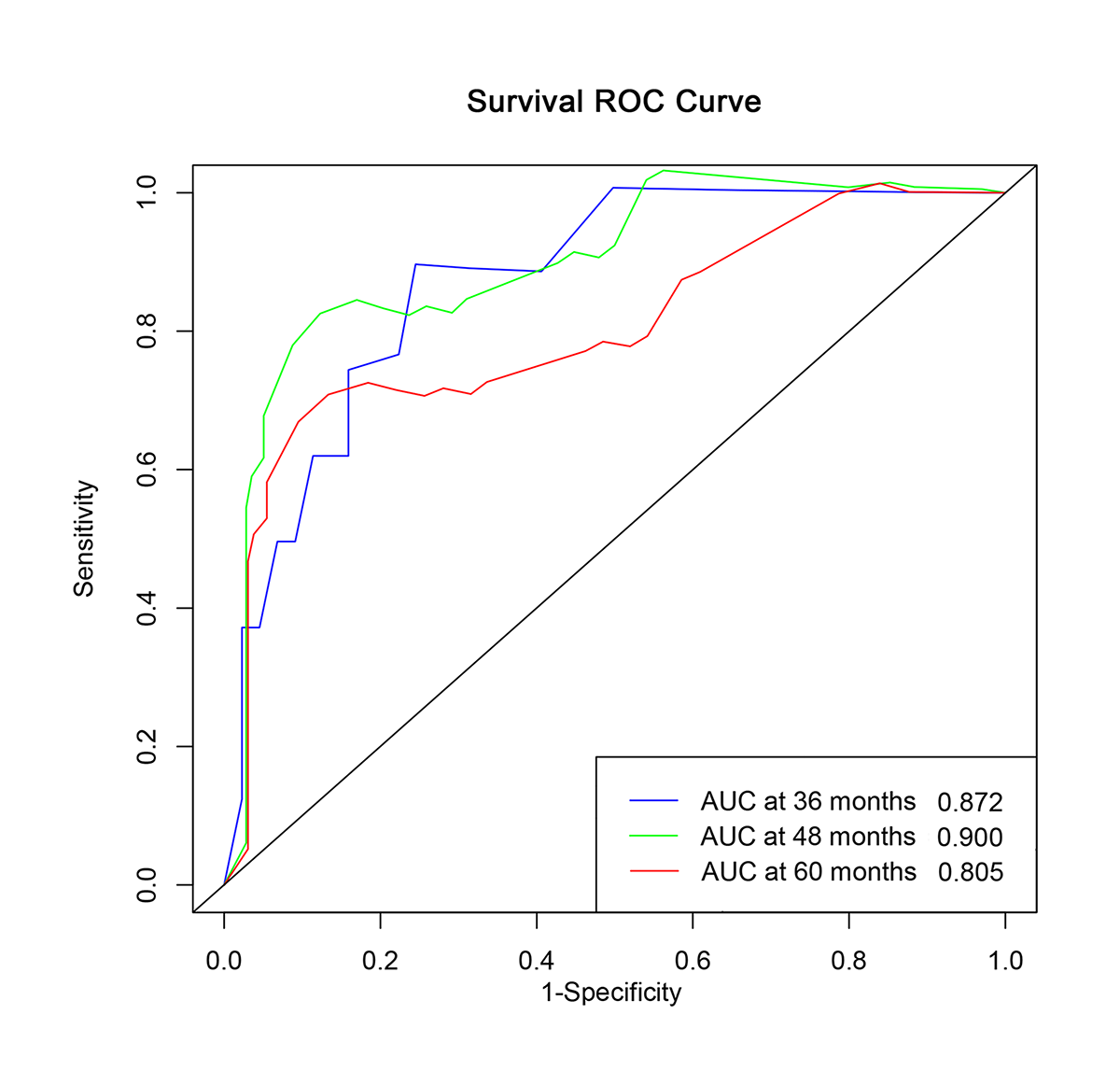

Supplement: Supplementary Figure 2 — Time-dependent ROC curves based on the timing of surgery in the delayed group. [file Image_2.tif]
