# Supplementary material for: Prophylactic inguinal lymphadenectomy for high-risk cN0 penile cancer: The optimal surgical timing
Source: Front Oncol. 2023 Feb 21;13:1069284. doi: 10.3389/fonc.2023.1069284 (PMC9989449; doi:10.3389/fonc.2023.1069284)
Supplement: Supplementary file 3 [file Table_1.docx]

**Table S1** Surgical outcomes of the immediate and delayed groups.

| Variable | Immediate (n=35) | Delayed (n=52) | *p* |
| --- | --- | --- | --- |
| Lymph nodes removed, n (median [IQR]) | | | |
| Left side | 9 (7, 11) | 8 (7, 10) | 0.554 |
| Right side | 8 (7, 9) | 8 (7, 10) | 0.518 |
| Total | 18 (15, 19) | 17 (15, 19) | 0.927 |
| Total number of positive lymph nodes, n (%) | 11 (1.8) | 68 (7.7) | ＜0.001* |
| Wound complications, n (%) | 5 (7.1) | 19 (18.3) | 0.037* |
| Wound infection | 2 (2.9) | 13 (12.5) | 0.026* |
| Skin necrosis | 2 (2.9) | 7 (6.7) | 0.317 |
| Lymphorrhea | 2 (2.9) | 10 (9.6) | 0.126 |
| Wound dehiscence | 1 (1.4) | 3 (2.9) | 0.649 |
| Clavien-Dindo classification, n (sides) (%) |  |  |  |
| I | 3 (4.3) | 4 (3.8) | 1.000 |
| II | 2 (2.9) | 6 (5.8) | 0.477 |
| IIIa | 0 | 6 (5.8) | 0.082 |
| IIIb | 0 | 3 (2.9) | 0.274 |
| Patient with positive lymph node, n (%) | 6 (17.1) | 28 (53.8) | 0.001* |
| Pathological lymph node status, n (%) |  |  | 0.001* |
| pN0 | 29 (82.9) | 24 (46.2) |  |
| pN1 | 4 (11.4) | 11 (21.2) |  |
| pN2 | 1 (2.9) | 15 (28.8) |  |
| pN3 | 1 (2.9) | 2 (3.8) |  |
| IQR, inter-quartile range. *p* values are derived from two-tailed tests. *All differences statistically significant at *p*＜0.05. | | | |
