# Supplementary material for: Prophylactic inguinal lymphadenectomy for high-risk cN0 penile cancer: The optimal surgical timing
Source: Front Oncol. 2023 Feb 21;13:1069284. doi: 10.3389/fonc.2023.1069284 (PMC9989449; doi:10.3389/fonc.2023.1069284)
Supplement: Supplementary file 4 [file Table_2.docx]

**Table S2** Surgical outcomes of the early and late groups.

| Variable | Early (n=34) | Late (n=18) | *p* |
| --- | --- | --- | --- |
| Lymph nodes removed, n (median [IQR]) | | | |
| Left side | 8 (7, 10.25) | 7.5 (7, 9.25) | 0.187 |
| Right side | 8 (7, 10) | 8.5 (7, 11) | 0.605 |
| Total | 17 (15.75, 19) | (15, 19.25) | 0.861 |
| Total number of positive lymph nodes, n (%) | 24 (4.1) | 44 (14.6) | ＜0.001* |
| Wound complications, n (%) | 6 (8.8) | 13 (36.1) | 0.001* |
| Wound infection | 4 (5.9) | 9 (25.0) | 0.010* |
| Skin necrosis | 2 (2.9) | 5 (13.9) | 0.047* |
| Lymphorrhea | 3 (4.4) | 7 (19.4) | 0.030* |
| Wound dehiscence | 1 (1.5) | 2 (5.6) | 0.274 |
| Clavien-Dindo classification, n (%) |  |  |  |
| I | 3 (4.4) | 1 (2.8) | 1.000 |
| II | 2 (2.9) | 4 (11.1) | 0.179 |
| IIIa | 1 (1.5) | 5 (13.9) | 0.018* |
| IIIb | 0 | 3 (8.3) | 0.039* |
| Patient with positive lymph node, n (%) | 14 (41.2) | 14 (77.8) | 0.012* |
| Pathological lymph node status, n (%) |  |  | 0.002* |
| pN0 | 20 (58.8) | 4 (22.2) |  |
| pN1 | 8 (23.5) | 3 (16.7) |  |
| pN2 | 6 (17.6) | 9 (50.0) |  |
| pN3 | 0 | 2 (11.1) |  |
| IQR, inter-quartile range. *p* values are derived from two-tailed tests. *All differences statistically significant at *p*＜0.05. | | | |
