# Supplementary material for: Prophylactic inguinal lymphadenectomy for high-risk cN0 penile cancer: The optimal surgical timing
Source: Front Oncol. 2023 Feb 21;13:1069284. doi: 10.3389/fonc.2023.1069284 (PMC9989449; doi:10.3389/fonc.2023.1069284)
Supplement: Supplementary file 5 [file Table_3.docx]

**Table S3** Complications of the OILND in immediate and delayed groups.

| Variable | Immediate (44 sides) | Delayed (56 sides) | *p* |
| --- | --- | --- | --- |
| Wound complications, n (%) | 4 (9.1) | 16 (28.6) | 0.016* |
| Wound infection | 2 (4.5) | 11 (19.6) | 0.026* |
| Skin necrosis | 2 (4.5) | 7 (12.5) | 0.292 |
| Lymphorrhea | 1 (2.3) | 9 (16.1) | 0.040* |
| Wound dehiscence | 1 (2.3) | 3 (5.4) | 0.628 |
| Clavien-Dindo classification, n (%) |  |  |  |
| I | 1 (2.3) | 3 (5.4) | 0.628 |
| II | 3 (6.8) | 4 (7.1) | 1.000 |
| IIIa | 0 | 6 (10.7) | 0.033* |
| IIIb | 0 | 3 (5.4) | 0.253 |
| OILND, open inguinal lymph nodes dissection. *p* values are derived from two-tailed tests. *All differences statistically significant at *p*＜0.05. | | | |
