# Supplementary material for: Prophylactic inguinal lymphadenectomy for high-risk cN0 penile cancer: The optimal surgical timing
Source: Front Oncol. 2023 Feb 21;13:1069284. doi: 10.3389/fonc.2023.1069284 (PMC9989449; doi:10.3389/fonc.2023.1069284)
Supplement: Supplementary file 6 [file Table_4.docx]

**Table S4** Complications of OILNDs in early and late groups.

| Variable | Early (38 sides) | Late (18 sides) | *p* |
| --- | --- | --- | --- |
| Wound complications, n (%) | 6 (15.8) | 10 (55.6) | 0.002* |
| Wound infection | 4 (10.5) | 7 (38.9) | 0.027* |
| Skin necrosis | 2 (5.3) | 5 (27.8) | 0.029* |
| Lymphorrhea | 3 (7.9) | 6 (33.3) | 0.024* |
| Wound dehiscence | 1 (2.6) | 2 (11.1) | 0.239 |
| Clavien-Dindo classification, n (%) |  |  |  |
| I | 3 (7.9) | 0 | 0.544 |
| II | 2 (5.3) | 2 (11.1) | 0.587 |
| IIIa | 1 (2.6) | 5 (27.8) | 0.011* |
| IIIb | 0 | 3 (16.7) | 0.029* |
| OILND, open inguinal lymph nodes dissection. *p* values are derived from two-tailed tests. *All differences statistically significant at *p*＜0.05. | | | |
